# The Nexus Between Sperm Membrane Integrity, Sperm Motility, and DNA Fragmentation

**DOI:** 10.3390/membranes15040109

**Published:** 2025-04-02

**Authors:** Alfredo Góngora, Stephen Johnston, Pablo Contreras, Carmen López-Fernández, Jaime Gosálvez

**Affiliations:** 1 Centro de Fertilidad Humana en Mexico DF, Ciudad de México 06760, Mexico; agongora@centrodefertilidad.com; 2 School of Environment, University of Queensland, Gatton, QLD 4343, Australia; 3 School of Veterinary Science, University of Queensland, Gatton, QLD 4343, Australia; 4 Halotech DNA, Parque Cientifico de Madrid, 28049 Madrid, Spain; pablo.contreras@halotechdna.com (P.C.); mariadelcarmen.lopez@uam.es (C.L.-F.); 5 Department of Biology, Universidad Autónoma de Madrid, 28049 Madrid, Spain; jaime.gosalvez@uam.es

**Keywords:** sperm plasma membrane integrity, sperm motility, sperm DNA fragmentation, cluster analysis, integrated sperm quality

## Abstract

This study investigated the interrelationships between sperm plasma membrane integrity, motility, and DNA fragmentation (SDF) to provide a more holistic understanding of male fertility. A total of 1159 ejaculates were analyzed for sperm membrane integrity (% dead spermatozoa), motility (% immotile spermatozoa), and SDF (% sperm with fragmented DNA). The statistical methods included non-parametric correlation analysis and artificial intelligence (AI)-generated cluster analysis to identify patterns based on these three parameters. The results showed a moderate correlation (ρ = 0.65; *p* < 0.000) between sperm membrane integrity and motility, indicating that immotile sperm were more likely to exhibit membrane damage. A weak correlation (ρ = 0.21; *p* < 0.000) suggested that DNA damage was largely independent of the other sperm parameters. Cluster analysis identified three main clusters: Cluster 0: high levels of low membrane integrity, immotile sperm, and moderate DNA fragmentation. Cluster 1: moderate membrane integrity and motility but extremely high DNA fragmentation. Cluster 2: the lowest levels of membrane damage, immotile sperm, and DNA fragmentation, indicating overall better sperm quality. The clustering techniques demonstrated their ability to integrate multiple sperm parameters, enabling a more individualized fertility diagnosis and potentially enhancing male infertility assessments.

## 1. Introduction

When a semen analysis reveals a problem in the functional characteristics of an ejaculate, multiple parameters are typically involved, albeit with varying levels of intensity. Consequently, the ejaculates of individuals are subsequently classified as normozoospermic, oligozoospermic, teratozoospermic, azoospermic, or various combinations of these categories [1]. Despite these specific classifications, there are nevertheless certain sperm parameters that demonstrate a degree of interdependence with each other. For example, sperm membrane quality and sperm motility are clearly inter-related [2,3]. In contrast, sperm DNA fragmentation (SDF) appears to be independent of initial sperm plasma membrane issues but can become more affected with further post ejaculatory processing or “in vitro” incubation as the sperm plasma membrane deteriorates [4].

The sperm plasma membrane is a fluid bi-lipid layer punctuated with proteins and ion channels that regulate ion and molecule transport, some of which are also essential for motility [5,6]. Mechanisms that can disrupt the sperm plasma membrane may, therefore, impact motility. Such factors include anisosmotic cellular environments, adverse chemical agents, changes in pH, thermal fluctuations, and oxidative stress, all of which increase membrane fluidity, destabilizing the plasma membrane architecture [7]. Damage to the cell membrane also gives rise to elevated intracellular concentrations of calcium ions and ROS, resulting in cellular swelling and death [8]. Such imbalances in redox equilibrium, with an excess of pro-oxidant molecules in the surrounding media, can compromise the sperm plasma membrane function, impair motility, and reduce sperm viability [9,10].

Possession of an intact plasma membrane is essential for fertilization as non-viable (dead) spermatozoa are incapable of fertilizing the oocyte, at least under natural conditions of non-assisted forms of reproduction. Simultaneous assessment of sperm plasma membrane integrity and motility helps to distinguish sperm that are immotile but still retain an intact plasma membrane from those that are dead. Failure to acknowledge this level of differentiation may result in errors in predicting pregnancy, especially when intracellular sperm injection (ICSI) of the oocyte is conducted as the primary fertilization strategy.

SDF is an inherent property of sperm production and maturation in individuals and can be readily detected in the initial ejaculate. However, some spermatozoa present in the initial ejaculate have an underlying susceptibility to DNA damage, which can only be revealed with further in vitro incubation, which is referred to as cryptic DNA damage [11]. This DNA damage is typically exposed after ejaculation, primarily as a sign of iatrogenic sperm damage. Consequently, sperm DNA quality assessed immediately after ejaculation and liquefication does not always correlate strongly with other sperm parameters [12,13].

However, once the sperm plasma membrane degrades post ejaculation, the genetic material is exposed to external insults that may compromise DNA integrity. Under these conditions, the presence of a pro-oxidant environment [10] or the direct actions of nucleases present in the seminal plasma [14] can result in DNA damage and indirectly trigger DNA degradation mechanisms, such as apoptosis [15].

In this study, we firstly investigate the degree of interdependence of the individual sperm parameters of plasma membrane integrity, motility, and DNA fragmentation, as assessed 30 min after ejaculation, by conducting a series of retrospective correlation analyses mined from a large patient database from a Mexican fertility clinic. Secondly, we present the results of semen evaluation as an integrated interpretation of the three sperm parameters, accommodating a holistic representation of their inter-relationships, rather than the traditional singular characterizations of semen quality.

## 2. Materials and Methods

### 2.1. Patients and Experimental Design

The respective relationships between individual patients’ measurements of sperm plasma membrane integrity (% dead sperm), sperm motility (% immotile sperm), and SDF (% fragmented sperm) were analyzed from a total of 1159 different patients attending the Centro de Fertilidad Humana in Mexico, Mexico City, Mexico. The ejaculates were all analyzed following WHO recommendations as part of the patients’ initial evaluation; none of the semen samples were used for fertilization. The project was approved by the clinic’s ethics committee (Reference: CF950302) and complies with fundamental ethical principles and standards of good clinical practice.

Once the semen sample was collected by masturbation, it was allowed to liquify at room temperature for a period of 30 min before evaluation. Sperm motility was assessed using CASA system SCA (Sperm Class Analyzer; Microptic, Barcelona, Spain) computer software and immotile spermatozoa were defined as those showing no physical movement or displacement. Sperm plasma membrane integrity was evaluated using Duo-Vital (HalotechDNA, Madrid, Spain) for fluorescence microscopy and the results were reported as the percentage of sperm with a compromised sperm plasma membrane. SDF was determined using the sperm chromatin dispersion test (Halosperm G2; HalotechDNA, Madrid, Spain) according to the manufacturer’s recommendations. During the human sperm chromatin dispersion test, sperm with a halo were indicative of those with intact DNA, whereas those without a halo were shown to have fragmented DNA.

**Figure 1:**
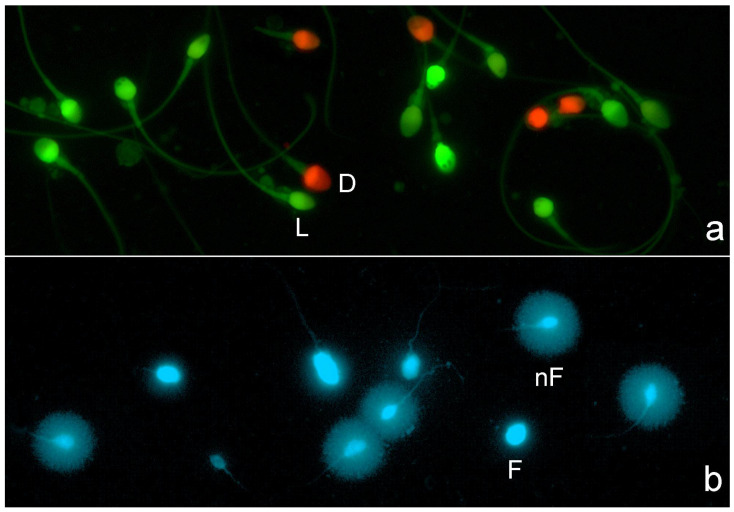
Fluorescence microscopy for visualization of sperm plasma membrane integrity and sperm DNA fragmentation. (**a**) Sperm plasm membrane integrity: red spermatozoa represent dead spermatozoa or spermatozoa with a damaged plasma membrane (D), whereas green spermatozoa represent live sperm (L) or spermatozoa with an intact plasm membrane. (**b**) Sperm DNA fragmentation: spermatozoa presenting a large halo of chromatin dispersion represent normal sperm with an absence of DNA fragmentation (nF), whereas spermatozoa with no halos represent spermatozoa with a fragmented DNA molecule (F).

All images were visualized and captured using a Nikon Eclipse microscope equipped with a high-resolution Nikon 12-bit CCD (Nikon DS-Q) and using a 40x fluorite objective. For sperm plasma membrane integrity image visualization, a double pass excitation filter-block was used. For image capture, separate channel visualization (red visualization and green visualization), double grey image file capture, and merging were used; red spermatozoa corresponded to those with damaged or compromised membranes (Figure 1a). For SDF staining, DAPI (4′,6-diamidino-2-phenylindole; Thermo-Fisher Scientific, Madrid, Spain) was used. Spermatozoa resulting in large halos of chromatin dispersion around a defined core were identified as presenting a non-fragmented DNA molecule, while those with a small or absent halo contained a fragmented DNA molecule (Figure 1b).

### 2.2. Statistical Analysis

Kolmogorov–Smirnov analysis was used to test for the normal distribution of the initial sperm parameter dataset. Correlation analysis was performed using non-parametric tests (Spearman’s rho). To compare each of the three sperm parameters with each other in a pair-wise manner, a Friedman test was used, and a Wilcoxon test was used to compare possible differences between each variable. All comparisons were performed using SPSS software (IBM SPSS Statistics, v.29, Armonk, NY, USA). The level of significance for statistical analysis was set at α = 0.05.

The statistical analyses, including cluster analysis and discriminant analysis, were conducted using Python (v3.11). Specifically, the following libraries were utilized: scikit-learn (v1.3.1) for statistical modelling, matplotlib (v3.8.0) and seaborn (v0.12.2) for data visualization, and pandas (v2.1.0) for data manipulation and preprocessing. Additionally, ChatGPT-4 (OpenAI) was employed to assist in the development of Python scripts and to streamline graphic generation.

For cluster analysis, the Elbow and Silhouette methods [16] were used to determine the optimal number of clusters that could be created using the following variables: percentage of immotile spermatozoa, percentage of sperm with a damaged plasma membrane, and percentage of spermatozoa with fragmented DNA. K-Means cluster analysis was selected as the algorithm for creating different clusters. Finally, to predict an individual’s membership in a specific cluster, discriminant functions derived from linear discriminant analysis (LDA) were employed.

## 3. Results

**Figure 2:**
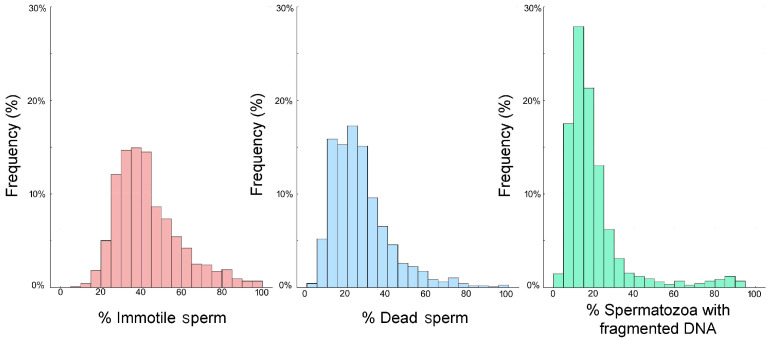
The frequency distributions of the % immotile spermatozoa (pink), % sperm with a damaged plasma membrane (blue), and % SDF (green) assessed from 1159 males visiting a Mexican fertility clinic.

Table 1 and Figure 2 report the descriptive statistics and frequency distribution of all three sperm parameters. The results of the Kolmogorov–Smirnov analysis determined that the datasets were not normally distributed, so non-parametric tests were employed. Table 1 shows that the percentage of immotile spermatozoa was higher than the percentage of spermatozoa with a damaged plasma membrane, whereas SDF had the lowest percentage of the three variables.

Significant differences were observed when the values associated with the three variables were compared (Friedman test, Chi^2^ = 1,493,701; *p* < 0.000). The percentage of immotile spermatozoa was significantly higher (Z = −27.98; *p* < 0.000) than the percentage of spermatozoa with a damaged plasma membrane, and the percentage of spermatozoa with a damaged plasma membrane was significantly higher (Z = −17.465; *p* < 0.000) than the percentage of spermatozoa showing fragmented sperm DNA. Consequently, the percentage of immotile spermatozoa was also significantly higher (Z = −25.531; *p* < 0.000) than that of spermatozoa with fragmented DNA.

There was a moderate positive correlation between the percentage of immotile spermatozoa and the percentage of spermatozoa with a damaged plasma membrane (ρ = 0.65; *p* < 0.000), suggesting that samples with a higher percentage of immotile spermatozoa also tended to have a higher percentage of plasma membrane-damaged spermatozoa. The correlation between % immotile spermatozoa and % SDF was low (ρ = 0.21; *p* < 0.000), similar to the correlation observed between sperm with a damaged plasma membrane and the percentage of fragmented spermatozoa (ρ = 0.21; *p* < 0.000).

**Figure 3:**
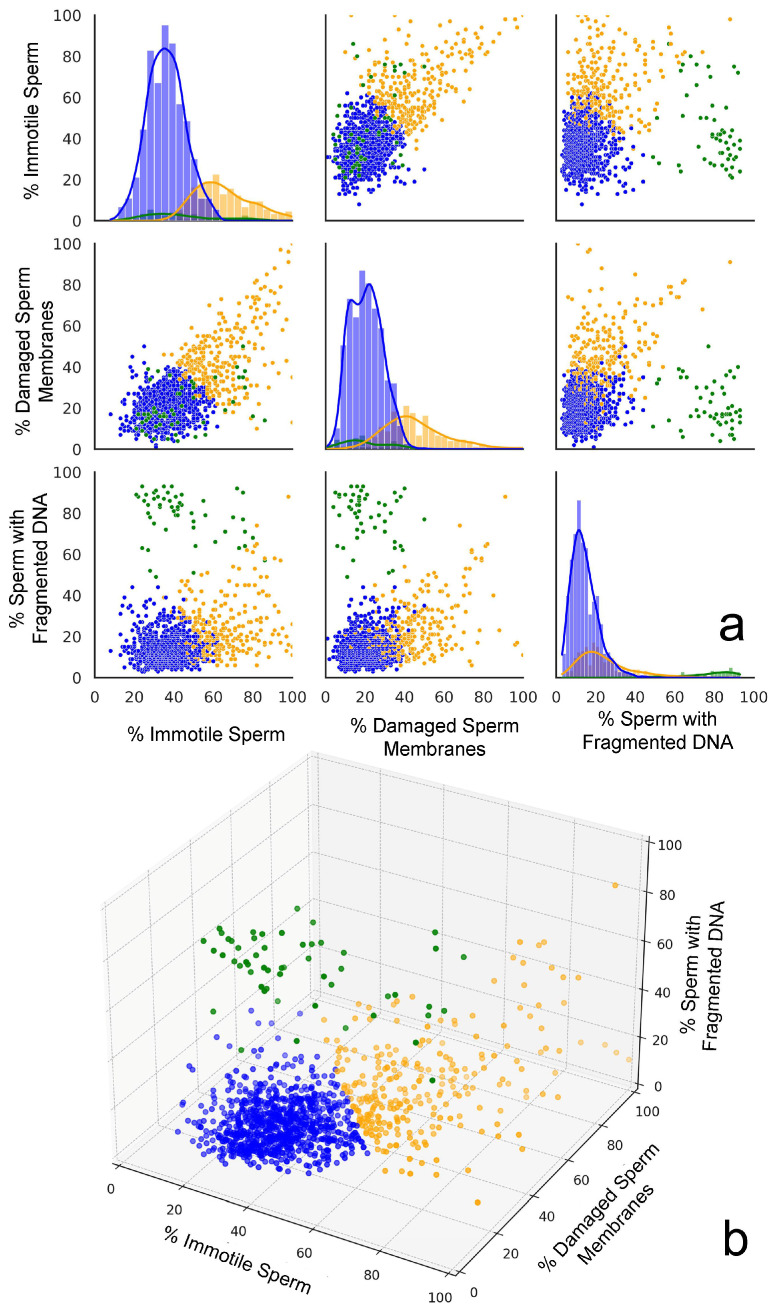
A graphic representation of the three plots generated from cluster analysis. (**a**) Scatter plot matrices displaying the relationships between the percentages of immotile spermatozoa, spermatozoa with a damaged plasma membrane, and sperm with fragmented DNA across the three distinct clusters identified in the dataset. The scatter plots indicate distinctive clustering patterns. The diagonal histograms represent the distribution of each sperm parameter (immotile, damaged membranes, and fragmented DNA) within each cluster. Each cluster is represented by a different color: Cluster 0 (orange), Cluster 1 (green), and Cluster 2 (blue). (**b**) The 3D scatter plot provides a comprehensive view of how the three sperm parameters interact simultaneously, revealing the spatial separation of clusters in the parameter space.

The Elbow method and the Silhouette score for the optimal number of clusters were used to determine the number of clusters that could be established; both methods suggested that three clusters might be optimal for this dataset. Clustering approaches were performed using K-Means (Silhouette score: 0.50; Davies–Bouldin score: 0.82), DBSCAN (Silhouette score: 0.36; Davies–Bouldin score: 1.72), and agglomerative clustering (Silhouette score: 0.45; Davies–Bouldin score: 0.86). Based on the evaluation metrics, K-Means appeared to be the best clustering method for this dataset, providing the most well-defined clusters with the highest Silhouette score and lowest Davies–Bouldin score. K-Means offered a good balance between cluster cohesion and separation, which made it a suitable choice for further analysis. Two- and three-dimensional representations of the clustering performed using the three variables are shown in Figure 3a,b, respectively.

The cluster analysis identified three distinct groups in the data. Cluster 0 (orange cloud in Figure 3a) was formed by 260 individuals’ semen samples and is characterized by high percentages of immotile spermatozoa (64.9%) and spermatozoa with a damaged plasma membrane (45.4%), along with a moderately elevated percentage of spermatozoa with fragmented DNA (24.2%). Cluster 1 (green cloud in Figure 3a) consists of samples from 54 individuals whose semen samples exhibit moderate levels of immotile spermatozoa (43.8%) and SDF (20.4%), but are distinguished by extremely high percentages of sperm with fragmented DNA (78.9%). Cluster 2 (blue cloud in Figure 3a) was formed by samples from 845 individuals whose semen samples are characterized by the lowest percentage of immotile spermatozoa (36.2%), as well as spermatozoa with a damaged plasma membrane (20.6%) and spermatozoa with fragmented DNA (14.4%).

**Figure 4:**
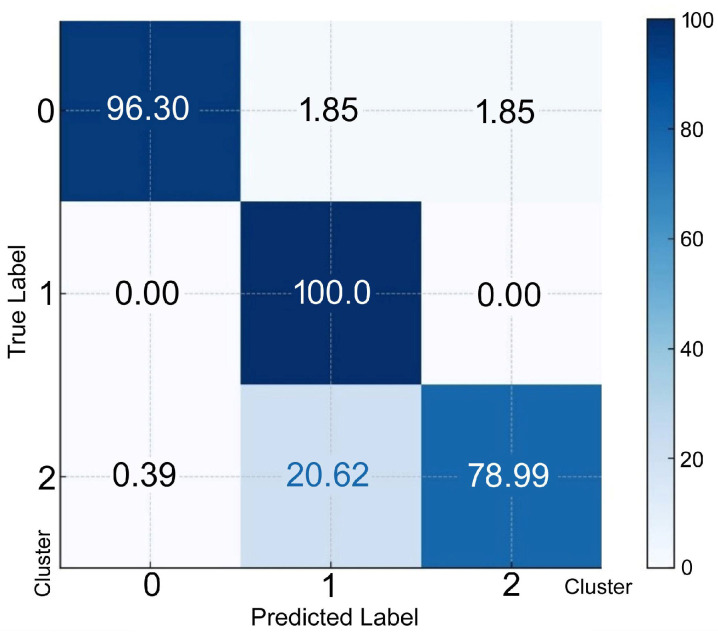
The confusion matrix (percentage) for discriminant analysis. The rows represent the actual (true) classes from the dataset. The X-axis represents the predicted classes generated by the discriminant analysis model. The true label on the Y-axis indicates the percentage of instances of correct classification as Cluster 0, 1, or 2.

 To predict an individual’s membership in a specific cluster, discriminant functions derived from LDA were used. Each discriminant function computed a score for each cluster and the cluster with the highest score was selected as the predicted cluster. The confusion matrix for discriminant analysis, as represented in percentages, is shown in Figure 4 Cluster 0 resulted in 96.3% of the individuals being correctly classified; 1.85% and 1.85% were misclassified as clusters 1 and 2, respectively. Cluster 1 resulted in 100.00% correctly classified individuals. Cluster 2 resulted in 79.0% of individuals being classified correctly; 0.39% and 20.62% were misclassified as clusters 0 and 1, respectively.

## 4. Discussion

The results of this study indicate that human sperm membrane integrity (26.1% dead), sperm motility (42.9% immotile), and SDF (19.5%) are relatively imprecise predictors with respect to each other, at least when assessed immediately following liquification 30 min after ejaculation. Although a proportion of immotile spermatozoa can still possess an intact sperm membrane, there is nonetheless a moderate correlation between the proportion of immotile spermatozoa and those with a damaged plasma membrane. By contrast, the proportion of sperm with fragmented DNA shows a low level of correlation with the proportions of both immotile spermatozoa and spermatozoa with a damaged plasma membrane. In addition, the means observed for the proportions of immotile spermatozoa and those with a damaged sperm plasma membrane were higher than that of SDF. While the relationship between sperm motility and plasma membrane integrity is somewhat self-evident and has a basis in the literature, the relationship between sperm motility and SDF is less established and, therefore, much more tenuous.

The mechanisms that promote sperm movement are complex and involve multiple physiological and biochemical processes [17]. Adenosine triphosphate (ATP) provides the energy for supporting the key functions of spermatozoa, including motility, and is formed by two metabolic pathways: glycolysis and oxidative phosphorylation. ATP is produced in the mitochondria through oxidative phosphorylation but also by glycolysis in the head and the principal piece of the flagellum [18]. Ion channels linked to calcium, potassium, and sodium ions can also modulate the activity of dynein arms and the flagellar beat pattern, thereby impacting motility [19,20]. All these functions can be disrupted when the membranes are functionally modified. The important role of sperm membrane lipids in membrane fluidity must also be considered, as they act independently of serum lipid levels and have been implicated as independent markers of reproductive function [21].

Although the specific relationship between membrane integrity and sperm motility has not been investigated in detail, observations from the current study are consistent with those described in other species. For example, stallion sperm motility has been shown to decline more rapidly compared to membrane damage following cooled storage [22] and even boar spermatozoa show a similar trend when exposed to anisosmotic events [23]. Thus, it is not uncommon for sperm motility values to be lower than those associated with membrane quality. Such differential effects may also be exacerbated when spermatozoa are challenged post ejaculation because the impact of iatrogenic damage can adversely affect the fluidity of the plasma membrane.

The sperm membrane can be compromised when there is an increase in its permeability that results in leakage of essential cellular components and ions [5,24]. In parallel, the lipid bilayer may undergo changes such as lipid oxidation, which affects membrane fluidity and stability [25]. Lipids in the sperm membrane clearly play a key role in sperm integrity because they act independently of serum lipid levels, and these changes are so evident that they have been referred to as independent markers of reproductive function [25]. The sperm membrane is also composed of polyunsaturated fatty acids, which increase the sperm cell’s susceptibility to oxidative stress. Oxidative stress may increase sperm protein oxidation via carbonylation, impacting membrane protein function (membrane receptors and channels) and ultimately impairing motility [26]. A loss of membrane integrity also fundamentally alters the sperm cell’s capacity to undergo a range of physiological processes associated with fertility, such as capacitation and the acrosome reaction.

Although plasma membrane integrity, sperm motility, and SDF are all likely to be affected synergistically by multiple factors including oxidative stress, age, lifestyle factors, smoking, alcohol consumption, obesity, exposure to environmental toxins, infections, medical conditions, heat exposure, nutritional deficiencies, and stress [27], it is also possible to attribute an increase in SDF with related loss of sperm motility, membrane disruption, and oxidative stress [10] to the action of seminal plasma DNases [14].

### Towards a Holistic View of the Seminogram

Given the differing levels of correlation observed among the three sperm parameters used in the current experiment and the difficulty in correlating the three variables from a functional viewpoint, we attempted to analyze the variables by means of a cluster analysis. This type of investigation presents an assessment of sperm quality from an individual patient as an integrated function of all variables, leading to their allocation into specific subpopulations. From a clinical perspective, this synergistic interactive approach in which multiple parameters are considered may, with further refinement and perhaps with the inclusion of even more variables, give rise to new categorizations for each patient that may better reflect their underlying pathology.

Presently, patients are classified as asthenozoospermic (reduced sperm motility), oligozoospermic (low sperm count), teratozoopermic (high amount of abnormal sperm morphology), azoospermic (no spermatozoa present in the ejaculate), or a combination of these, but the interactions between these different classifications of sperm quality are rarely considered. In the future, a different strategy to classify patients according to the relative weight of different sperm parameters (e.g., sperm motility, sperm with damaged membranes, or SDF) could be used to produce higher-order levels of characterization and discrimination for each individual. As an example of such an integrative assessment in the present analysis, Cluster 0 was formed by 260 individuals characterized by high percentages of immotile (64.9%) and dead sperm 45.4%, along with a moderately elevated percentage of SDF (24.2%). The elevated percentages of immotile spermatozoa and spermatozoa with a damaged plasma membrane suggest significant impairment in sperm motility and membrane viability. Although the percentage of fragmented sperm DNA was notable, it was lower than that found in Cluster 1, indicating that while DNA integrity was compromised, the primary issues were associated with sperm motility and the sperm plasma membrane. These individuals should be considered suitable for ICSI, while the possibility of performing IU or IVF is excluded. To reduce the level of SDF, the implementation of advanced sperm selection methodologies that effectively lower SDF levels in the sample is recommended. This could be achieved, in the first instance, by using a standard protocol such as swim up (SU) or density gradient centrifugation (DGC) but later combining different techniques for more specific sperm selection, such as magnetic activated cell sorting (MACS), high-magnification microscopy (IMSI), or physiological ICSI (PICSI).

In another scenario, Cluster 1 consisted of 54 individuals who exhibited moderate levels of immotile spermatozoa (43.8%) and spermatozoa with a damaged plasma membrane (20.4%) but were distinguished by an extremely high percentage of SDF (78.9%). The defining feature of this cluster was the severe degree of DNA fragmentation, which surpassed that of the other clusters, and which has a critical impact on sperm functionality. Despite moderate sperm motility and spermatozoa with a damaged plasma membrane, the high level of DNA damage would have serious implications for fertility. These patients might correspond to those identified in a normal seminogram as idiopathic and can be considered as candidates for sperm selection procedures in order to remove the majority of spermatozoa with fragmented DNA, as described above.

Finally, Cluster 2 was formed by 845 individuals presenting the lowest percentages of immotile spermatozoa (36.2%), spermatozoa with a damaged plasma membrane (20.6%), and SDF (14.4%). This cluster is indicative of higher overall sperm quality, with lower levels of dysfunction across all the measured parameters. The relatively low percentages of immotile spermatozoa and those with a damaged plasma membrane, along with minimal DNA fragmentation, suggest that individuals in this cluster may have the highest potential for successful fertilization and reproductive outcomes. In contrast to the individuals classified in the previous clusters, these individuals can be regarded as good candidates for IU, IVF, or ICSI.

The dataset used for the current analysis was only based on a static assessment of the ejaculate. Given that SDF can also be determined as a dynamic process where SDF varies in a time-dependent manner [4,28], our present analysis may be further refined and improved by the incorporation of additional variables that also involve changes in sperm motility, sperm plasma membrane integrity, and SDF.

We propose that traditional methods of diagnosing male infertility, such as semen analysis considering isolated variables, are limited in their ability to provide a full picture of male reproductive health. While variables such as sperm concentration, sperm motility, sperm morphology, and semen volume are often analyzed separately in traditional semen analysis, an integrative model examines how these parameters interact with each other to provide a more nuanced patient-centric understanding of their fertility.

In addition, the information provided by omics approaches can be combined with classical semen analysis data to provide an even deeper understanding of male reproductive health and identify specific molecular markers that might explain abnormalities in conventional semen parameters [29,30]. Other variables obtained from an analysis of the seminal plasma can also be integrated [31]. The quantity of information provided through this integrative approach, in principle at least, is unlimited because its complexity can be readily managed using AI algorithms to establish the relative load of each variable and generate a specific cluster.

We see a future in which AI-generated information can provide greater insight into fertility, since different sperm parameters might be assigned different weights of importance based on their known impact on fertility. For example, in procedures incorporating ICSI, sperm DNA fragmentation or the presence of certain enzymes (e.g., DNase [4]) might carry more weight than semen volume or sperm motility. Similarly, high sperm counts but low motility could indicate different fertility outcomes compared with a low sperm count accompanied by high motility. Teratospermia, abnormal morphology combined with SDF, could be indicative of more severe fertility issues [32].

By integrating multiple parameters, an individual’s fertility status can be classified into risk categories (e.g., low, moderate, and high risk of infertility). The integrative model might generate a fertility index score, which provides a general assessment of the likelihood of successful conception based on the weighted average of sperm parameters. This will provide a more complete landscape for each individual to predict natural conception outcomes or the success rates of assisted reproductive techniques. An integrative semen analysis is likely to lead to a personalized fertility diagnosis and this, in turn, leads to more tailored and efficacious recommendations for treatment.

## Figures and Tables

**Table 1:** The descriptive statistics of the static analysis of % immotile spermatozoa, % sperm with a damaged plasma membrane (SDPM), and % SDF from 1159 individuals.

	Mean	SD	Lower Quartile	Median	Upper Quartile
% Immotile	42.9	16.3	31	40	51
% SDPM	26.1	14.7	16	23	33
% SDF	19.5	16.3	16	15	21

## Data Availability

The raw data supporting the conclusions of this article will be made available by the authors on request.
